# Seasonality of composition, genomic potential and activity of coniferous forest soil microbiomes

**DOI:** 10.1038/s41597-026-07163-w

**Published:** 2026-04-23

**Authors:** Zander Rainier Human, Martina Štursová, Iñaki Odriozola, Tomáš Větrovský, Adina Howe, Diana Navrátilová, Rubén López-Mondéjar, Lucia Žifčáková, Vendula Brabcová, Sunil Mundra, Ella Thoen, Luis Morgado, Anna Maria Fiore-Donno, Michael Bonkowski, Bartosz Adamczyk, Petr Kohout, Mary S. Lipton, Sara Calhoun, Kurt LaButti, Anna Lipzen, Keykhosrow Keymanesh, Sravanthi Tejomurthula, Christa Pennacchio, Igor V. Grigoriev, Francis Martin, Håvard Kauserud, Petr Baldrian

**Affiliations:** 1 Laboratory of Environmental Microbiology, Institute of Microbiology of the Czech Academy of Sciences, Vídeňská 1083, Praha, 14200 Czech Republic; 2 Aquatic Geomicrobiology, Institute of Biodiversity, Ecology and Evolution, Friedrich Schiller University Jena, Dornburger Strasse 159, Jena, 07743 Germany; 3 Department of Agricultural and Biosystems Engineering, Iowa State University, Ames, IA USA; 4 Okinawa Institute of Science and Technology, Physics and Biology Unit, 1919-1 Tancha, Onna-son, Kunigami-gun, Okinawa, 904-0495 Japan; 5 Department of Biology, College of Science, United Arab Emirates University, Al-Ain, Abu Dhabi UAE; 6 Khalifa Center for Genetic Engineering and Biotechnology, United Arab Emirates University, Al-Ain, Abu Dhabi UAE; 7 Section for Genetics and Evolutionary Biology (EvoGene), Department of Biosciences, University of Oslo, Oslo, Norway; 8 Naturalis Biodiversity Center, Leiden, The Netherlands; 9 Terrestrial Ecology Group, Institute of Zoology, University of Cologne, Cologne, Germany; 10 Cluster of Excellence on Plant Sciences (CEPLAS), Cologne, Germany; 11 Natural Resources Institute Finland (Luke), Latokartanonkaari 9, Helsinki, FI 00790 Finland; 12 Faculty of Science, Charles University, Albertov 6, Prague, 128 40 Czechia; 13 Environmental Molecular Sciences Laboratory, Pacific Northwest National Laboratory, Richland, WA 99352 United States of America; 14 Department of Energy Joint Genome Institute, Lawrence Berkeley National Laboratory, Berkeley, CA USA; 15 Department of Plant and Microbial Biology, University of California Berkeley, Berkeley, CA USA; 16 Environmental Genomics and Systems Biology Division, Lawrence Berkeley National Laboratory, Berkeley, CA USA; 17 Université de Lorraine, INRAE, UMR Interactions Arbres/Micro-organismes, Centre INRAE Grand-Est Nancy, 54280 Champenoux, France; 18 The National Key Laboratory of Ecological Security and Sustainable Development in the Arid Region, Northwest Institute of Eco-Environment and Resources, Chinese Academy of Sciences, 730000 Lanzhou, China

**Keywords:** Soil microbiology, Microbial ecology

## Abstract

Coniferous forest soils represent a globally important carbon sink, where the microbiome is essential for carbon flux between tree roots, rhizosphere, litter and soil. Soil habitats, such as roots, rhizosphere, bulk soil and litter differ in physicochemical properties and composition of highly specialized microbial communities, whose activity reflects the seasonality of temperature and tree activity of these mid- to high-latitude biomes. Here we present a multi-omic dataset encompassing 160 samples collected from four coniferous forest soil habitats in the Czech Republic and Norway, sampled in early summer, late summer, early winter and late winter that characterize the composition, genomic potential and activity of tree roots and microbiome. For each sample, we provide metabarcoding-based composition of bacterial, fungal and eukaryotic communities, results of shotgun DNA sequencing (metagenomes) and shotgun RNA sequencing (metatranscriptomes) illustrating the functional potential and activity within habitats. This dataset enables analyses of the temporal variation of taxonomic composition, functional potential and transcription across seasons in a temperate and boreal coniferous forest.

## Background & Summary

Temperate and boreal coniferous forests represent one of the largest terrestrial carbon sinks^1,2^, with soil microbial communities driving the utilization, fixation and long‐term storage of organic carbon^3–5^. These soils exhibit spatial and functional heterogeneity, where specific, discrete habitats, including plant roots, rhizosphere soil, bulk soil and litter can be distinguished, each characterised by distinct resource inputs and microbial processes^6,7^. Tree roots and the surrounding rhizosphere are rich in ectomycorrhizal (ECM) fungi, with mycelial networks extending from root tips through the rhizosphere, creating hotspots of microbial activity^5,7,8^. In bulk soil, ECM fuelled by root-derived carbon decompose organic matter in search of N and P^9^ alongside saprotrophic fungi and bacteria, which prevail in the absence of ECM^10–12^. Litter, essentially dead plant biomass is the main source of organic matter in topsoil heavily colonized by saprotrophic fungi^13^, many of which are specialized to decompose cellulose, hemicelluloses and lignin^14^, and bacteria, both decomposers and opportunists^15^.

Tree roots and their nutrient inputs are also major drivers of seasonality in forest soil microbial communities, beyond the obvious seasonal effects of temperature and snow cover that are better understood. Changes in root phenology such as regrowth in early summer^16^, peak exudation in late summer^17,18^ and the absence of continuous input of root‐allocated C in winter, drive compartment‐specific changes in microbial biomass, community composition, and biogeochemical activity^7^. Previous studies in coniferous forest soils often only compared summer versus winter^19,20^ and focused on bulk soil and litter^6,21^, while others focused on root and rhizosphere microbiomes without considering the interplay with other compartments^5,12,22^. By sampling across early summer, late summer, early winter, and late winter, we can distinguish tree-driven phenological effects (early vs. late summer) from abiotic seasonality (winter vs. summer) and reveal how temporal variation in root activity and environmental conditions jointly shape microbial community dynamics and biogeochemical functioning of soil microhabitats.

In this Data Descriptor, we present comprehensive sequencing data from plant roots, rhizosphere, bulk soil, and litter in *Picea abies* forests in the Czech Republic and Norway. For each country, we sampled four habitats across five plots and four seasonal time points (early summer, late summer, early winter, late winter), yielding 80 samples per country and 160 samples in total (4 habitats × 5 plots × 4 seasons × 2 countries). DNA-derived data include metabarcodes of bacterial 16S rRNA, fungal internally transcribed spacer (ITS2), and universal eukaryotic and cercozoan-specific 18S rRNA amplicons and shotgun metagenomes from all 160 samples. In addition, we present metatranscriptomes from RNA co-extracted from the same samples and depleted of rRNA, which have already been assembled, annotated and re-aligned to annotated assemblies. To explore the activity of the keystone microbes in this ecosystem, the ECM and ericoid mycorrhizal fungi, we present fungal genomes from the studied experimental sites. Finally, to accompany sequencing data, we provide information on soil and litter chemistry, fungal biomass content and bacterial-to-fungal ribosomal copy number ratios.

## Methods

### Study area and sample collection

**Fig. 1:**
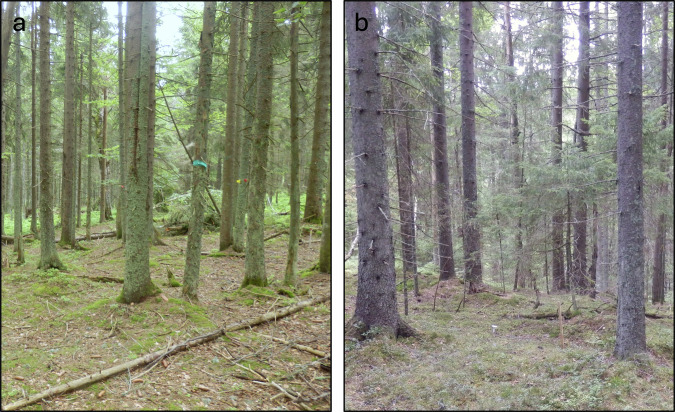
Czech sampling plots located near Přílba in the Bohemian Forest Mountain range were dominated by *Picea abies* and had a sparse understory of grasses (*Avenella*, *Calamagrostis*), bilberry (*Vaccinium myrtillus*), and mosses. Norwegian sampling plots (**b**) near Maridalen, Oslo, were also *P. abies*-dominated but with a denser understory of grasses, ericoid shrubs (*Vaccinium* spp.), and mosses.

Sample locations and protocols were previously described in detail^23,24^. We sampled two Norway spruce (*Picea abies*) forests. The first site was in the Bohemian Forest, Czech Republic (49°02′ N, 13°37′ E; Fig. 1a), at 1170–1200 m a.s.l., where the long-term mean annual temperature is 5.0 °C and mean annual precipitation is about 1000 mm. The second site was near Oslo, Norway (59°59′ N, 10°47′ E; Fig. 1b), at 170–180 m a.s.l., with a mean annual temperature of 5.8 °C and an average annual precipitation of 680 mm. In each forest, five sampling plots spaced roughly 250 m apart were established and revisited at 4 month intervals. Sampling covered four successive seasons, early summer (June/July), late summer (September, peak vegetation period), early winter (December/January) and late winter (March/April). GPS coordinates of all plots, sampling dates and associated soil and litter chemistry are summarized in Table 1, with additional plot-level metadata provided in Table S1^25^.

**Table 1:** Mean (±SE) of soil and litter chemistry from Norwegian and Czech sampling plots. Site-specific chemistry is available in Table S1, as previously reported^23^.

	Soil		Litter	
	Czech Rep.	Norway	Czech Rep.	Norway
**pH (H**_**2**_**O)**	3.36 ± 0.03	3.76 ± 0.04	3.50 ± 0.02	4.09 ± 0.10
**pH (KCl)**	2.82 ± 0.03	3.19 ± 0.06	2.92 ± 0.01	3.43 ± 0.11
**N (%)**	1.23 ± 0.07	1.02 ± 0.15	1.83 ± 0.07	1.76 ± 0.04
**C (%)**	29.17 ± 1.03	19.40 ± 2.24	50.33 ± 1.43	47.64 ± 1.39
**C org. (%)**	23.57 ± 3.37	16.37 ± 3.41	47.42 ± 1.32	44.92 ± 1.57
**C-carbonate (%)**	5.60 ± 2.45	3.03 ± 1.16	2.91 ± 1.29	2.72 ± 0.92
**NH**_**4**_^**+**^ **(mg.kg**^**−1**^**)**	12.53 ± 1.55	17.42 ± 2.16	13.93 ± 5.31	41.19 ± 31.31
**NO**_**3**_^**-**^ **(mg.kg**^**−1**^**)**	29.66 ± 2.37	24.37 ± 3.36	25.81 ± 5.16	62.76 ± 17.14
**N-NH**_**4**_^**+**^ **(mg.kg**^**−1**^**)**	9.73 ± 1.20	13.52 ± 1.68	10.82 ± 4.13	31.98 ± 24.31
**N-NO**_**3**_^**-**^ **(mg.kg**^**−1**^**)**	6.70 ± 0.54	5.50 ± 0.76	5.83 ± 1.16	14.18 ± 3.87
**Ca (mg.kg**^**−1**^**)**	235.4 ± 46.2	219.4 ± 32.9	503.83 ± 114.18	1338.30 ± 323.70
**Mg (mg.kg**^**−1**^**)**	115.65 ± 30.52	128.98 ± 86.39	219.78 ± 39.72	492.36 ± 131.28
**K (mg.kg−**^**1**^**)**	171.4 ± 17.0	136.8 ± 13.5	490.31 ± 137.52	736.62 ± 109.06
**P (extractable) (mg.kg**^**−1**^**)**	13.8 ± 3.0	10.4 ± 3. 8	72.02 ± 31.34	106.69 ± 38.08
**CEC (mmol.kg**^**−1**^**)**	182.9 ± 7.7	171.8 ± 18.8	232.47 ± 14.56	261.44 ± 21.47

Sampling was conducted on predefined 3 ×3 m plots by taking 16 soil cores (4 cm diameter). All material from the litter layer down to bottom of the organic horizon was collected and separated into plant roots, litter, bulk soil and rhizosphere soil. Litter was cut to pieces smaller than 0.5 cm, rhizosphere and bulk soil separately passed through a 5 mm sieve, and plant roots immediately washed in sterile, RNase-free water and cut into smaller pieces. Samples were immediately frozen in liquid nitrogen and transported on dry ice before storage at −80 °C.

Fungal biomass in rhizosphere, soil and litter samples was determined as the content of ergosterol extracted from freeze-dried material using 10% KOH in methanol as previously described^26^ and quantified by high-performance liquid chromatography (HPLC) (Table S2^25^). Chitin content was measured as glucosamine released after acid hydrolysis of chitin, followed by derivatization using 9-fluorenylmethyl-chloroformate (FMOC-Cl). Samples were measured using HPLC (Arc HPLC, Waters, USA) equipped with a Hewlett Packard ODS Hypersil (5 μm, 250 × 4.6 mm) column and detected via fluorescence detector^27^.

### Soil and litter chemistry

Soil and litter physicochemical properties were measured from powdered soil and litter samples by an external laboratory and was previously reported in detail^23^. To measure pH, powdered samples were mixed with 5 mL of either deionized water or potassium chloride (KCl), and measured using a WTW Multilab 540 pH meter. Total N and C content were determined through dry combustion at 1000 °C in a pure oxygen environment using a Flash 2000 elemental analyzer (Thermo Scientific). Organic carbon and carbonate content were estimated via a separate analysis using acid decomposition with hydrochloric acid and subsequent quantification of carbon dioxide released by fumigation. Nitrate and ammonium content were extracted with 0.5 M potassium sulfate and quantified using a Quikchem FIA 8000 flow injection analyser (Lachat Instruments) operated with Omnion 3.0 software. Exchangeable calcium (Ca), potassium (K), and magnesium (Mg) were extracted using the Mehlich II method and their concentrations were measured via atomic absorption spectrophotometry using a ContrAA 700 instrument (Analytik Jena).

### DNA and RNA extraction

Samples were flash frozen in liquid nitrogen, crushed and homogenized with a mortar and pestle immediately followed by extraction. Total RNA was extracted from 1 g aliquots of rhizosphere soil, bulk soil and litter samples in 3 replicates per sample using the RNeasy PowerSoil RNA extraction kit (Qiagen). Co-extracted DNA was collected using the RNeasy Powersoil DNA Elution kit (Qiagen). Extracted RNA and DNA were purified and pooled using OneStep PCR Inhibitor Removal Kit (Zymo Research). RNA from roots was extracted and purified using the NucleoSpin RNA Plant Kit (Macherey Nagel) and DNA extracted using a DNeasy Plant Maxi kit (Qiagen) from 250 mg aliquots in 3 replicates per sample. The concentration and purity of extracted DNA and RNA were checked using Qubit and Nanodrop. Replicate DNA and RNA extracts from each sample were pooled before further analysis.

DNA was removed from RNA extracts through a DNase digest and its successful removal was confirmed using a 16S rRNA PCR reaction using universal primers 515F and 806R^28^ giving negative amplification results. Ribosomal RNA was depleted from purified RNA extracts using a combination of Ribo-Zero rRNA removal kit human/mouse/rat and Ribo-Zero rRNA removal kit bacteria (Illumina) as described previously^29^. RNA quality and integrity was confirmed using an Agilent 2100 Bioanalyzer.

### Amplicon sequencing

Bacterial communities were characterised by amplifying the V4 region of the 16S rRNA using primers 515F and 806R^29 ^ and fungal communities by amplifying internally transcribed spacer 2 (ITS2) using primers gITS7 and ITS4^30^ using conditions previously reported^19^. Microbial eukaryotic communities were amplified using primers TAReuk454FWD and TAReukREV3^31^ using a protocol previously published^32^. Cercozoan communities were previously sequenced by amplification of the V4 region of the 18S rRNA gene using Cercozoa-specific primers and reported in an earlier publication^24^. They originate from the same samples and are reported here for completeness.

All PCR reactions were performed in triplicate and subsequently pooled and purified using a Qiagen MinElute purification kit (Qiagen). Library preparation was performed using a TruSeq DNA PCR-Free LT Kit (Illumina) and sequenced on an Illumina MiSeq (2 × 250 bp).

Bacteria/fungi ratios were determined by quantification of bacterial and fungal rRNA gene copies using qPCR using the 1108 f and 1132r primers for bacteria^33,34^ and FR1 and FF390 primers for fungi^35^. The bacteria/fungi ratio was calculated by dividing respective rRNA gene copy numbers.

### Metagenomic and metatranscriptomic sequencing

Metagenomes from late summer samples (n = 40) were generated at the Department of Energy (DOE) Joint Genome Institute using the Illumina HiSeq-2000 1TB platform (2 × 151 bp).

Metagenomes were processed according to a DOE JGI Metagenome workflow previously described^36^. BBDuk (version 37.22) from the BBTools software package^37^ (Bushnell, 2014; https://jgi.doe.gov/data-and-tools/bbtools/) was used to remove contaminants, trim reads that contained adapter sequence and low-quality trailing bases, cutting reads at the first base with a Phred quality score of 0. It was also used to remove reads with 1 or more ‘N’ bases, with average quality score across the read less than 10, and reads that, after trimming, were shorter than 51 bp or <33% of their original untrimmed read length. Reads that mapped with BBMap (from BBTools^37^) to human, cat, dog and mouse reference sequences were considered as contamination and also removed. In addition, common microbial contaminants such as *E. coli*, several *Klebsiella, Shigella* genomes, *Stenotrophomonas maltophilia*, *Ralstonia solanacearum Staphylococcus aureus* and others (Table S3^25^) were removed prior to assembly. The number of reads identified as microbial contaminants ranged from 32–3582, which represented 0.0001–0.05% of the total reads per sample. Contaminants identified as human, dog, cat or mouse origin were also negligible, ranging from 0–0.1% of total reads per sample. Filtered reads were assembled using metaSPADES 3.11.1^38^. Filtered reads were mapped back to contigs larger than 200 bp using BBMap 38.44 with the output saved to a coverage file. Assemblies were annotated using the DOE-JGI Metagenome Annotation Pipeline (MAP) version 4.16.5^39^. Identification of protein-coding genes was performed with Prodigal v2.6.3^40^ and GeneMark v2.8^41^ after CRISPR arrays and non-coding RNA genes were identified. Protein products were functionally annotated with KEGG Orthology^42^, Pfam^43^, TIGRfam^44^, COG^45^ and InterPro Scan^46^.

Metatranscriptome reads from all 160 samples were processed in the same way as metagenomes, except that ribosomal RNA reads were removed using rqcfilter.sh script, also part of the BBTools package^37^, prior to assembly using MEGAHIT v1.0.6^47^. Assembled metatranscriptomes were then annotated using the DOE-JGI Metagenome Annotation Pipeline (MAP) version 4.16.5 as described for metagenomes^39^. Quality filtered rRNA depleted reads were mapped to metatranscriptome assemblies using BBMap^37^, and coverage statistics for mapping is available on the JGI Genome Portal^48^.

To complement the 40 metagenomes from late-summer samples sequenced by the JGI, we produced additional metagenomes for early summer, early winter and late winter (n = 120) samples in the authors’ laboratories. Sample processing and DNA extractions were done using the same protocols as for the late-summer metagenomes. Sequencing libraries from litter and bulk soil and rhizosphere samples were constructed using a TruSeq DNA PCR-Free kit (Illumina, San Diego, CA, USA), and root metagenomic libraries were constructed using a TruSeq DNA Nano kit (Illumina). The only difference was that the 40 late-summer metagenomes were sequenced on an Illumina HiSeq platform, whereas the additional 120 metagenomes were sequenced on an Illumina NovaSeq 6000. Sequence data are made available in ‘fastq’ format without any further processing.

### Fungal genome sequencing

Sporocarps of the ectomycorrhizal fungi *Russula emetica*, *Boletus edulis*, *Amanita rubescens*, *Russula ochroleuca* were collected at Přilba forest in the Bohemian forest mountain range where the soil samples from this study were collected. Genome sequencing data, assembly, annotation and analysis of these genomes have already been published^49^. Sporocarps of the ectomycorrhizal fungi *Elaphomyces granulatus*, *Lactarius tabidus*, *Russula decolorans* and *Tylopilus felleus* were collected from Maridalen, Norway from the same location as Norwegian soil samples. The ericoid mycorrhizal fungus *Hyaloscypha variabilis* was isolated from the roots of *Vaccinium* sp. from the same location. DNA was extracted using a CTAB method^50^, as suggested in the JGI 1000 Fungal genomes protocol^51^ and RNA was extracted using NucleoSpin RNA Plant and Fungi kit (Macherey-Nagel).

Fungal genomes were sequenced using a PacBio Sequel IIe platform. A total of 1.5 µg of genomic DNA per sample was sheared to a target fragment size of ~10 kb using the Megaruptor 3 (Diagenode), treated with exonuclease, DNA damage repair enzyme mix and end-repair/A-tailing mix and ligated with SMRTbell Barcoded Adapter Plate 3.0 (PacBio) using SMRTbell Express Template Prep Kit 2.0 (PacBio). Libraries were purified with AMPure PB Beads (PacBio), pooled to achieve 1.5 Gb of PacBio CCS sequence and size-selected using the 0.75% agarose gel cassettes with Marker S1 and High Pass protocol on the BluePippin (Sage Science). Sequencing primer was then annealed to the SMRTbell template library and sequencing polymerase bound using the Sequel II Binding kit 2.0. Libraries were then sequenced with SMRT Link 10.2, 8 M v1 SMRT cells, v2.0 chemistry and 1×1800 movie times.

Mitochondrial genomes were assembled by separating CCS reads likely belonging to organelles from nuclear genome reads using coverage and GC filtering (cutoffs: GC < 0.40; coverage < 1.5× k-mer peak using BBTools v.38.79 (kmercountexact.sh with default settings, bbnorm.sh with passes=1, bits=16, target=9999999 min=58 and bbduk.sh with maxgc=0.4). Flye version 2.9^52^ (-g 100k–asm-coverage 100–pacbio-hifi) was used for an initial assembly. Genes were predicted with Prodigal v2.6.3^40^, and screened using HMMER^53^ against a custom database of mitochondrial HMMs. This database was constructed by building HMMs for mitochondrial gene families from manually curated multiple sequence alignments of corresponding proteins in NCBI’s RefSeq mitochondrial proteomes, with outliers and misannotated sequences removed prior to HMM construction. Contigs where putative mitochondrial genes were identified had ribosomal loci masked (bases replaced with ‘N’ characters) using BBTools^37^ (bbduk.sh with settings k=25 mm=f kmask=N) using SILVA v138.1^54^ as reference database. The masked contigs were then used to recruit additional CCS reads using BBtools^37^ (k=25 mm=f mkf=0.03 ordered ow). Recruited reads assembled with Flye^52^ after which another round of read recruitment and assembly was performed. Contigs <1 kb were excluded and the final assembly was polished with two rounds of RACON^55^ version 1.4.13 [-u -t 36]. The mitochondria-filtered CCS reads were then also assembled with Flye^52^ and polished with two rounds of RACON version 1.4.13^55^.

Fungal transcriptomes were sequenced using Illumina RNASeq with PolyA Selection using the TruSeq Stranded mRNA HT kit (Illumina). Each library was prepared from 1 µg RNA per sample and amplified with 8 PCR cycles. Libraries were quantified with the Illumina KAPA library quantification kit (Roche) using a LightCycler 480 (Roche). Libraries were pooled and sequenced on an Illumina NovaSeq6000 (2 × 150 bp indexed run).

Raw reads from fungal transcriptomes were processed using the JGI QC pipeline. BBDuk (from BBTools^37^) was used to detect artifact sequences by kmer matching (kmer = 25), allowing 1 mismatch and trimming detected artifact sequences from the 3’ end of the reads. PhiX, reads containing Ns and reads with average quality below Q6 were also removed using BBDuk and post-QC reads shorter than 25 bases or below 1/3 of the original read length were also removed. Quality filtered fastq files were used as input for de novo assembly of RNA contigs using Trinity v2.11.0^56^. Genomes were annotated using the JGI Annotation pipeline^49^ and both Illumina RNASeq and PacBio IsoSeq.

## Data Records

Soil and litter chemistry for all sites sampled in this study are summarised in Table 1, and complete site-level data are provided in Table S1^25^. Fungal biomass and ribosomal copy number ratios are provided in Table S2^25^. A complete summary of all sequencing data, including 16S rRNA, ITS2, Cercozoan- and universal 18S rRNA, metagenome and metatranscriptome data, including NCBI run numbers with accompanying metadata is available in Table S4^25^. All supplementary data is available on figshare (10.6084/m9.figshare.31314859)^25^. Amplicon sequencing data is provided in ‘fastq’ format with sample barcodes and sequencing adapters removed. 16S rRNA and ITS2 sequencing data are available from the NCBI Sequence Read Archive (SRA) under the accession PRJNA1075501^57^ for samples from the Czech Republic, and PRJNA1260938^58^ for samples from Norway. 18S rRNA data from the Czech Republic and Norway are available under PRJNA1260921^59^. In addition to these datasets, Cercozoan-specific 18S rRNA sequences generated in a previous study^24^, but from the same samples, are available under PRJNA776131^60^. Read files for metagenomes from Czech and Norwegian sampling sites are deposited in the NCBI SRA under the BioProject accessions listed in Table 2, BioProject, SRR and SRP accessions are listed in Table S4^25^ and as citations^61–101^. All run numbers on a per-sample basis are listed in Table S4^25^ to enable easy downloadable access to data.

**Table 2:** NCBI SRA BioProject accession numbers for all sequencing data presented here.

Dataset	Country	Bioproject accession
16S rRNA	Czech Republic	PRJNA1075501
16S rRNA	Norway	PRJNA1260938
ITS2	Czech Republic	PRJNA1075501
ITS2	Norway	PRJNA1260938
Universal 18S rRNA	Czech Republic	PRJNA1260921
Universal 18S rRNA	Norway	PRJNA1260921
Cercozoa 18S rRNA	Czech Republic	PRJNA776131
Cercozoa 18S rRNA	Norway	PRJNA776131
Metagenomes	Czech Republic	PRJNA467677 - PRJNA467691, PRJNA468121 - PRJNA468124, PRJNA502406, PRJNA1233945
Metagenomes	Norway	PRJNA570524 - PRJNA570528, PRJNA502715 - PRJNA502729, PRJNA1233945
Metatranscriptomes	Czech Republic	PRJNA538294–PRJNA538296, PRJNA468259–PRJNA468298, PRJNA444105–PRJNA444126, PRJNA443929–PRJNA443941, PRJNA519841, PRJNA465698
Metatranscriptomes	Norway	PRJNA622083–PRJNA622086, PRJNA622036, PRJNA621582, PRJNA537844–PRJNA537858, PRJNA519914–PRJNA519972

Comprehensive sample accession numbers are listed in Table S4^25^.

Metatranscriptomes from the Czech and Norwegian sampling sites have also been deposited to the NCBI SRA under the BioProject accessions listed in Table 2, BioProject, SRR and SRP accession listed in Table S4^25^ and as citations^102–261^.

The genomes of five fungi collected from the sites in Norway were sequenced and have been submitted to the NCBI and are available under accessions JBTAFJ000000000^262^, JBTIYH000000000^263^, JBTYLQ000000000^264^, JBTYLS000000000^265^ and JBTYLR000000000^266^ (Table 3). Four fungal genomes from the Czech sites were previously sequenced and published^49^ and all associated data are also available through the NCBI WGS Accessions WHVC00000000^267^, WIQF00000000^268^, WION00000000^269^, WHVB00000000^270^ (Table 3).

**Table 3:** Fungal genomes available from forests sampled in this study. Raw sequencing data, genome assemblies, transcriptomes and all genome annotations can be accessed at JGI Mycocosm portal^277^ using the abbreviation provided.

Fungus	Collection location	NCBI WGS Acc.	JGI Mycocosm
*Elaphomyces granulatus*	Maridalen, Norway	JBTAFJ000000000^262^	ElagrMar1^278^
*Lactarius tabidus*	Maridalen, Norway	JBTIYH000000000^263^	Lactab1^279^
*Russula decolorans*	Maridalen, Norway	JBTYLQ000000000^264^	RusdecM1^280^
*Tylopilus felleus*	Maridalen, Norway	JBTYLS000000000^265^	Tylfel1^281^
*Hyaloscypha variabilis*	Maridalen, Norway	JBTYLR000000000^266^	Hyavar1^282^
*Russula emetica*	Přilba, Czech Republic	WHVC00000000^267^	Ruseme1^49,283^
*Boletus edulis*	Přilba, Czech Republic	WIQF00000000^268^	Boledp1^49,284^
*Amanita rubescens*	Přilba, Czech Republic	WION00000000^269^	Amarub1^49,285^
*Russula ochroleuca*	Přilba, Czech Republic	WHVB00000000^270^	Rusoch1^49,286^

## Technical Validation

All samples were collected aseptically using sterilized equipment and stored in sterile nuclease free tubes. RNA and DNA were extracted in a clean, RNase free environment. The quantity and quality of extracted RNA and DNA were assessed in a Qubit 2.0 Fluorometer and Nanodrop device. RNA integrity and fragment size distributions were evaluated using an Agilent 2100 Bioanalyzer and DNA and RNA size distributions determined using agarose gel electrophoresis on a 1% agarose gel. DNA was removed from RNA samples using a DNase digestion and confirmed using a PCR reaction with bacterial 16S rRNA primers 515 F and 806 R.

All amplicon data were produced through PCR amplification with negative and positive controls. Amplification was confirmed using agarose gel electrophoresis, and amplicon concentrations measured using a Qubit 2.0 fluorometer. After processing raw sequencing data, reads containing ambiguous bases or with mean quality scores below 30 were omitted.

## Usage Notes

For 16S rRNA, ITS2, and universal and Cercozoa-specific 18S rRNA sequence data, quality filtered ‘fastq’ files are available through the NCBI SRA (Table 1, Table S4^25^), and can be used to infer sequence variants using pipelines such as DADA2^271^ or to perform clustering using programs such as SEED2^272^ or USEARCH^273^.

Raw sequence data for all JGI-sequenced samples are also available from the NCBI SRA in ‘fastq’ format (Table 2; Table S4^25^), including metatranscriptomes from all 160 samples, and 40 metagenomes from late summer sampling points. In addition, 120 metagenomes from early summer, and early and later winter are also available in ‘fastq’ format from the NCBI SRA.

In addition to the raw sequence data for all JGI-sequenced samples available on the NCBI SRA, data can also be accessed under study ID Gs0128948^274^ from the JGI Integrated Microbial Genomes and Metagenomes IMG/MER portal^275,276^, and can easily be accessed using the ‘search’ function. Under this study ID users can access sequencing data, assemblies and annotations. In addition, we provide JGI IMG/MER sample accessions for individual samples as downloadable supplementary table, which can also be accessed using the ‘search’ function on the JGI IMG/MER portal.

## Supplementary information

Table S1

Table S2

Table S3

Table S4

## Data Availability

All data reported in this study are publicly available. The eight amplicon sequencing, 160 metagenome and 160 metatranscriptome datasets are available from the NCBI Sequence Read Archive (SRA) under the accession numbers listed in Table S4, available along with all associated sample data from Figshare^25^. NCBI BioProject accession numbers for amplicon, metagenomic and metatranscriptomic datasets are listed in Table 2. Newly sequenced fungal genomes can be accessed from the NCBI through whole-genome shotgun accessions JBTAFJ000000000^262^, JBTIYH000000000^263^, JBTYLQ000000000^264^, JBTYLS000000000^265^ and JBTYLR000000000^266^.

## Code availability

No custom code was used to generate the data presented in this manuscript. The scripts used for processing metagenome and metatranscriptome sequence data are available at Figshare^25^ (10.6084/m9.figshare.31314859).

## References

[1] Pan, Y. *et al*. A large and persistent carbon sink in the world’s forests. *Science* **333**, 988–993, 10.1126/science.1201609 (2011). 21764754 10.1126/science.1201609

[2] Baldrian, P., López-Mondéjar, R. & Kohout, P. Forest microbiome and global change. *Nat. Rev. Microbiol.* **21**, 487–501, 10.1038/s41579-023-00876-4 (2023). 36941408 10.1038/s41579-023-00876-4

[3] Kramer, C. & Gleixner, G. Soil organic matter in soil depth profiles: distinct carbon preferences of microbial groups during carbon transformation. *Soil Biol. Biochem.* **40**, 425–433, 10.1016/j.soilbio.2007.09.016 (2008).

[4] Schmidt, M. W. I. *et al*. Persistence of soil organic matter as an ecosystem property. *Nature* **478**, 49–56, 10.1038/nature10386 (2011). 21979045 10.1038/nature10386

[5] Clemmensen, K. E. *et al*. Roots and associated fungi drive long-term carbon sequestration in boreal forest. *Science* **339**, 1615–1618, 10.1126/science.1231923 (2013). 23539604 10.1126/science.1231923

[6] Štursová, M., Bárta, J., Šantrůčková, H. & Baldrian, P. Small-scale spatial heterogeneity of ecosystem properties, microbial community composition and microbial activities in a temperate mountain forest soil. *FEMS Microbiol. Ecol.* **92**, fiw185, 10.1093/femsec/fiw185 (2016). 27604254 10.1093/femsec/fiw185

[7] Baldrian, P. Forest microbiome: diversity, complexity and dynamics. *FEMS Microbiol. Rev.* **41**, 109–130, 10.1093/femsre/fuw040 (2017). 27856492 10.1093/femsre/fuw040

[8] Lebeis, S. L. Greater than the sum of their parts: characterizing plant microbiomes at the community-level. *Curr. Opin. Plant Biol.* **24**, 82–86, 10.1016/j.pbi.2015.02.004 (2015). 25710740 10.1016/j.pbi.2015.02.004

[9] Smith, S. E. & Read, D. J. Mycorrhizal Symbiosis, 10.1016/B978-0-12-370526-6.X5001-6 (Academic Press, 2008).

[10] Van der Wal, A., Geydan, T. D., Kuyper, T. W. & De Boer, W. A thready affair: linking fungal diversity and community dynamics to terrestrial decomposition processes. *FEMS Microbiol. Rev.* **37**, 477–494, 10.1111/1574-6976.12001 (2013). 22978352 10.1111/1574-6976.12001

[11] Fernandez, C. W. & Kennedy, P. G. Revisiting the ‘Gadgil effect’: do interguild fungal interactions control carbon cycling in forest soils? *New Phytol.* **209**, 1382–1394, 10.1111/nph.13648 (2016). 26365785 10.1111/nph.13648

[12] Kohout, P. *et al*. Clearcutting alters decomposition processes and initiates complex restructuring of fungal communities in soil and tree roots. *ISME J.* **12**, 692–703, 10.1038/s41396-017-0027-3 (2018). 29335638 10.1038/s41396-017-0027-3 PMC5864242

[13] Lindahl, B. D. *et al*. Spatial separation of litter decomposition and mycorrhizal nitrogen uptake in a boreal forest. *New Phytol.* **173**, 611–620, 10.1111/j.1469-8137.2006.01936.x (2007). 17244056 10.1111/j.1469-8137.2006.01936.x

[14] Štursová, M. *et al*. Cellulose utilization in forest litter and soil: identification of bacterial and fungal decomposers. *FEMS Microbiol. Ecol.* **80**, 735–746, 10.1111/j.1574-6941.2012.01343.x (2012). 22379979 10.1111/j.1574-6941.2012.01343.x

[15] Lladó, S., Větrovský, T. & Baldrian, P. Tracking of the activity of individual bacteria in temperate forest soils shows guild-specific responses to seasonality. *Soil Biol. Biochem.* **135**, 275–282, 10.1016/j.soilbio.2019.05.010 (2019).

[16] Puhe, J. Growth and development of the root system of Norway spruce (Picea abies) in forest stands—a review. *For. Ecol. Manage.* **175**, 253–273, 10.1016/S0378-1127(02)00134-2 (2003).

[17] Högberg, M. N. *et al*. Quantification of effects of season and nitrogen supply on tree below-ground carbon transfer to ectomycorrhizal fungi and other soil organisms in a boreal pine forest. *New Phytol.* **187**, 485–493, 10.1111/j.1469-8137.2010.03274.x (2010). 20456043 10.1111/j.1469-8137.2010.03274.x

[18] Ekblad, A. *et al*. The production and turnover of extramatrical mycelium of ectomycorrhizal fungi in forest soils: role in carbon cycling. *Plant Soil* **366**, 1–27, 10.1007/s11104-013-1630-3 (2013).

[19] Žifčáková, L., Větrovský, T., Howe, A. & Baldrian, P. Microbial activity in forest soil reflects the changes in ecosystem properties between summer and winter. *Environ. Microbiol.* **18**, 288–301, 10.1111/1462-2920.13026 (2016). 26286355 10.1111/1462-2920.13026

[20] Žifčáková, L. *et al*. Feed in summer, rest in winter: microbial carbon utilization in forest topsoil. *Microbiome* **5**, 1–12, 10.1186/s40168-017-0340-0 (2017). 28923122 10.1186/s40168-017-0340-0 PMC5604414

[21] Baldrian, P. *et al*. Active and total microbial communities in forest soil are largely different and highly stratified during decomposition. *ISME J.* **6**, 248–258, 10.1038/ismej.2011.95 (2012). 21776033 10.1038/ismej.2011.95 PMC3260513

[22] Law, S. R. *et al*. Metatranscriptomics captures dynamic shifts in mycorrhizal coordination in boreal forests. *Proc. Natl Acad. Sci. USA* **119**, e2118852119, 10.1073/pnas.2118852119 (2022). 35727987 10.1073/pnas.2118852119 PMC9245616

[23] Starke, R. *et al*. Niche differentiation of bacteria and fungi in carbon and nitrogen cycling of different habitats in a temperate coniferous forest: a metaproteomic approach. *Soil Biol. Biochem.* **155**, 108170, 10.1016/j.soilbio.2021.108170 (2021).

[24] Fiore-Donno, A. M. *et al*. Soil compartments (bulk soil, litter, root and rhizosphere) as main drivers of soil protistan communities distribution in forests with different nitrogen deposition. *Soil Biol. Biochem.* **168**, 108628, 10.1016/j.soilbio.2022.108628 (2022).

[25] Human *et al*. Seasonality of composition, genomic potential and activity of coniferous forest soil microbiomes - Supplementary Data 10.6084/m9.figshare.31314859 (2026). 10.1038/s41597-026-07163-w PMC13315874 42026082

[26] Šnajdr, J. *et al*. Spatial variability of enzyme activities and microbial biomass in the upper layers of Quercus petraea forest soil. *Soil Biol. Biochem.* **40**, 2068–2075, 10.1016/j.soilbio.2008.01.015 (2008).

[27] Adamczyk, S. *et al*. An optimized method for studying fungal biomass and necromass in peatlands via chitin concentration. *Soil Biol. Biochem.* **149**, 107932, 10.1016/j.soilbio.2020.107932 (2020).

[28] Caporaso, J. G. *et al*. Global patterns of 16S rRNA diversity at a depth of millions of sequences per sample. *Proc. Natl Acad. Sci. USA* **108**, 4516–4522, 10.1073/pnas.1000080107 (2011). 20534432 10.1073/pnas.1000080107 PMC3063599

[29] Tláskal, V. *et al*. Complementary roles of wood-inhabiting fungi and bacteria facilitate deadwood decomposition. *mSystems* **6**, e01078–20, 10.1128/msystems.01078-20 (2021). 33436515 10.1128/mSystems.01078-20 PMC7901482

[30] Ihrmark, K. *et al*. New primers to amplify the fungal ITS2 region–evaluation by 454-sequencing of artificial and natural communities. *FEMS Microbiol. Ecol.* **82**, 666–677, 10.1111/j.1574-6941.2012.01437.x (2012). 22738186 10.1111/j.1574-6941.2012.01437.x

[31] Stoeck, T. *et al*. Multiple marker parallel tag environmental DNA sequencing reveals a highly complex eukaryotic community in marine anoxic water. *Mol. Ecol.* **19**, 21–31, 10.1111/j.1365-294X.2009.04480.x (2010). 20331767 10.1111/j.1365-294X.2009.04480.x

[32] Mundra, S. *et al*. Soil depth matters: shift in composition and inter-kingdom co-occurrence patterns of microorganisms in forest soils. *FEMS Microbiol. Ecol.* **97**, fiab022, 10.1093/femsec/fiab022 (2021). 33547899 10.1093/femsec/fiab022 PMC7948073

[33] Wilmotte, A., Van der Auwera, G. & De Wachter, R. Structure of the 16 S ribosomal RNA of the thermophilic cyanobacterium Chlorogloeopsis HTF (‘Mastigocladus laminosus’ HTF) strain PCC 7518, and phylogenetic analysis. *FEBS Lett.* **317**, 96–100, 10.1016/0014-5793(93)81499-P (1993). 8428640 10.1016/0014-5793(93)81499-p

[34] Amann, R. I., Ludwig, W. & Schleifer, K. H. Phylogenetic identification and *in situ* detection of individual microbial cells without cultivation. *Microbiol. Rev.* **59**, 143–169, 10.1128/mr.59.1.143-169.1995 (1995). 7535888 10.1128/mr.59.1.143-169.1995 PMC239358

[35] Prévost-Bouré, N. C. *et al*. Validation and application of a PCR primer set to quantify fungal communities in the soil environment by real-time quantitative PCR. *PLoS ONE* **6**, e24166, 10.1371/journal.pone.0024166 (2011). 21931659 10.1371/journal.pone.0024166 PMC3169588

[36] Clum, A. *et al*. DOE JGI metagenome workflow. *mSystems* **6**, e00804–20, 10.1128/mSystems.00804-20 (2021). 34006627 10.1128/mSystems.00804-20 PMC8269246

[37] Bushnell, B. BBMap. BMC Bioinformatics 13, 238, 10.1186/1471-2105-13-238 (2012).

[38] Nurk, S. *et al*. metaSPAdes: a new versatile metagenomic assembler. *Genome research* **27.5**, 824–834, 10.1101/gr.213959.116 (2017). 28298430 10.1101/gr.213959.116 PMC5411777

[39] Huntemann, M. *et al*. The standard operating procedure of the DOE-JGI metagenome annotation pipeline (MAP v. 4). *Stand. Genomic Sci.* **11**, 17, 10.1186/s40793-016-0138-x (2016). 26918089 10.1186/s40793-016-0138-x PMC4766715

[40] Hyatt, D. *et al*. Prodigal: prokaryotic gene recognition and translation initiation site identification. *BMC Bioinformatics* **11**, 119, 10.1186/1471-2105-11-119 (2010). 20211023 10.1186/1471-2105-11-119 PMC2848648

[41] Borodovsky, M., Mills, R., Besemer, J. & Lomsadze, A. Prokaryotic gene prediction using GeneMark and GeneMark.hmm. Curr. Protoc. Bioinform. 1, 4.5.1–4.5.5, 10.1002/0471250953.bi0405s01 (2003). 10.1002/0471250953.bi0405s01 18428700

[42] Kanehisa, M. *et al*. Data, information, knowledge and principle: back to metabolism in KEGG. *Nucleic Acids Res.* **42**, D199–D205, 10.1093/nar/gkt1076 (2014). 24214961 10.1093/nar/gkt1076 PMC3965122

[43] Punta, M. *et al*. The Pfam protein families database. *Nucleic Acids Res*. **40**, D290–D301 10.1093/nar/gkr1065 (2012). 10.1093/nar/gkr1065 PMC3245129 22127870

[44] Selengut, J. D. *et al*. TIGRFAMs and Genome Properties: tools for the assignment of molecular function and biological process in prokaryotic genomes. *Nucleic Acids Res.* **35**, D260–D264, 10.1093/nar/gkl1043 (2007). 17151080 10.1093/nar/gkl1043 PMC1781115

[45] Marchler-Bauer, A. *et al*. CDD: a conserved domain database for interactive domain family analysis. *Nucleic Acids Res.* **35**, D237–D240, 10.1093/nar/gkl951 (2007). 17135202 10.1093/nar/gkl951 PMC1751546

[46] Jones, P. *et al*. InterProScan 5: genome-scale protein function classification. *Bioinformatics* **30**, 1236–1240, 10.1093/bioinformatics/btu031 (2014). 24451626 10.1093/bioinformatics/btu031 PMC3998142

[47] Li, D. *et al*. MEGAHIT v1.0: a fast and scalable metagenome assembler driven by advanced methodologies and community practices. *Methods* **102**, 3–11, 10.1016/j.ymeth.2016.02.020 (2016). 27012178 10.1016/j.ymeth.2016.02.020

[48] DOE Joint Genome Institute Genome Portal. [http://genome.jgi.doe.gov/portal/].

[49] Miyauchi, S. *et al*. Large-scale genome sequencing of mycorrhizal fungi provides insights into the early evolution of symbiotic traits. *Nat. Commun.* **11**, 5125, 10.1038/s41467-020-18795-w (2020). 33046698 10.1038/s41467-020-18795-w PMC7550596

[50] Unruh, S. A. *et al*. Shallow genome sequencing for phylogenomics of mycorrhizal fungi from endangered orchids. Preprint at bioRxiv, 10.1101/862763 (2019).

[51] Grigoriev, I. V. *et al*. MycoCosm portal: gearing up for 1000 fungal genomes. *Nucleic Acids Res.* **42**, D699–D704, 10.1093/nar/gkt1183 (2014). 24297253 10.1093/nar/gkt1183 PMC3965089

[52] Kolmogorov, M., Yuan, J., Lin, Y. & Pevzner, P. A. Assembly of long, error-prone reads using repeat graphs. *Nat. Biotechnol.* **37**, 540–546, 10.1038/s41587-019-0072-8 (2019). 30936562 10.1038/s41587-019-0072-8

[53] Mistry, J., Finn, R. D., Eddy, S. R., Bateman, A. & Punta, M. Challenges in homology search: HMMER3 and convergent evolution of coiled-coil regions. *Nucleic Acids Res.* **41**, e121, 10.1093/nar/gkt263 (2013). 23598997 10.1093/nar/gkt263 PMC3695513

[54] Quast, C. *et al*. The SILVA ribosomal RNA gene database project: improved data processing and web-based tools. *Nucleic Acids Res.* **41**, D590–D596, 10.1093/nar/gks1219 (2013). 23193283 10.1093/nar/gks1219 PMC3531112

[55] Vaser, R., Sović, I., Nagarajan, N. & Šikić, M. Fast and accurate de novo genome assembly from long uncorrected reads. *Genome Res.* **27**, 737–746, 10.1101/gr.214270.116 (2017). 28100585 10.1101/gr.214270.116 PMC5411768

[56] Grabherr, M. G. *et al*. Full-length transcriptome assembly from RNA-seq data without a reference genome. *Nat. Biotechnol.* **29**, 644–652, 10.1038/nbt.1883 (2011). 21572440 10.1038/nbt.1883 PMC3571712

[57] *NCBI Sequence Read Archive* https://identifiers.org/ncbi/insdc.sra:SRP489333 (2025).

[58] *NCBI Sequence Read Archive* https://identifiers.org/ncbi/insdc.sra:SRP584133 (2025).

[59] *NCBI Sequence Read Archive* https://identifiers.org/ncbi/insdc.sra:SRP584119 (2025).

[60] *NCBI Sequence Read Archive* https://identifiers.org/ncbi/insdc.sra:SRP343709 (2025).

[61] *NCBI Sequence Read Archive* https://identifiers.org/ncbi/insdc.sra:SRP155162 (2025).

[62] *NCBI Sequence Read Archive* https://identifiers.org/ncbi/insdc.sra:SRP155168 (2025).

[63] *NCBI Sequence Read Archive* https://identifiers.org/ncbi/insdc.sra:SRP155169 (2025).

[64] *NCBI Sequence Read Archive* https://identifiers.org/ncbi/insdc.sra:SRP155171 (2025).

[65] *NCBI Sequence Read Archive* https://identifiers.org/ncbi/insdc.sra:SRP155176 (2025).

[66] *NCBI Sequence Read Archive* https://identifiers.org/ncbi/insdc.sra:SRP155177 (2025).

[67] *NCBI Sequence Read Archive* https://identifiers.org/ncbi/insdc.sra:SRP155188 (2025).

[68] *NCBI Sequence Read Archive* https://identifiers.org/ncbi/insdc.sra:SRP155189 (2025).

[69] *NCBI Sequence Read Archive* https://identifiers.org/ncbi/insdc.sra:SRP155190 (2025).

[70] *NCBI Sequence Read Archive* https://identifiers.org/ncbi/insdc.sra:SRP155194 (2025).

[71] *NCBI Sequence Read Archive* https://identifiers.org/ncbi/insdc.sra:SRP155195 (2025).

[72] *NCBI Sequence Read Archive* https://identifiers.org/ncbi/insdc.sra:SRP155201 (2025).

[73] *NCBI Sequence Read Archive* https://identifiers.org/ncbi/insdc.sra:SRP155202 (2025).

[74] *NCBI Sequence Read Archive* https://identifiers.org/ncbi/insdc.sra:SRP155205 (2025).

[75] *NCBI Sequence Read Archive* https://identifiers.org/ncbi/insdc.sra:SRP155208 (2025).

[76] *NCBI Sequence Read Archive* https://identifiers.org/ncbi/insdc.sra:SRP155211 (2025).

[77] *NCBI Sequence Read Archive* https://identifiers.org/ncbi/insdc.sra:SRP155212 (2025).

[78] *NCBI Sequence Read Archive* https://identifiers.org/ncbi/insdc.sra:SRP155213 (2025).

[79] *NCBI Sequence Read Archive* https://identifiers.org/ncbi/insdc.sra:SRP155223 (2025).

[80] *NCBI Sequence Read Archive* https://identifiers.org/ncbi/insdc.sra:SRP175151 (2025).

[81] *NCBI Sequence Read Archive* https://identifiers.org/ncbi/insdc.sra:SRP569563 (2025).

[82] *NCBI Sequence Read Archive* https://identifiers.org/ncbi/insdc.sra:SRP241047 (2025).

[83] *NCBI Sequence Read Archive* https://identifiers.org/ncbi/insdc.sra:SRP241045 (2025).

[84] *NCBI Sequence Read Archive* https://identifiers.org/ncbi/insdc.sra:SRP241041 (2025).

[85] *NCBI Sequence Read Archive* https://identifiers.org/ncbi/insdc.sra:SRP241039 (2025).

[86] *NCBI Sequence Read Archive* https://identifiers.org/ncbi/insdc.sra:SRP241038 (2025).

[87] *NCBI Sequence Read Archive* https://identifiers.org/ncbi/insdc.sra:SRP180776 (2025).

[88] *NCBI Sequence Read Archive* https://identifiers.org/ncbi/insdc.sra:SRP180775 (2025).

[89] *NCBI Sequence Read Archive* https://identifiers.org/ncbi/insdc.sra:SRP180774 (2025).

[90] *NCBI Sequence Read Archive* https://identifiers.org/ncbi/insdc.sra:SRP180773 (2025).

[91] *NCBI Sequence Read Archive* https://identifiers.org/ncbi/insdc.sra:SRP180772 (2025).

[92] *NCBI Sequence Read Archive* https://identifiers.org/ncbi/insdc.sra:SRP180771 (2025).

[93] *NCBI Sequence Read Archive* https://identifiers.org/ncbi/insdc.sra:SRP180770 (2025).

[94] *NCBI Sequence Read Archive* https://identifiers.org/ncbi/insdc.sra:SRP180588 (2025).

[95] *NCBI Sequence Read Archive* https://identifiers.org/ncbi/insdc.sra:SRP180587 (2025).

[96] *NCBI Sequence Read Archive* https://identifiers.org/ncbi/insdc.sra:SRP180586 (2025).

[97] *NCBI Sequence Read Archive* https://identifiers.org/ncbi/insdc.sra:SRP180585 (2025).

[98] *NCBI Sequence Read Archive* https://identifiers.org/ncbi/insdc.sra:SRP180584 (2025).

[99] *NCBI Sequence Read Archive* https://identifiers.org/ncbi/insdc.sra:SRP180581 (2025).

[100] *NCBI Sequence Read Archive* https://identifiers.org/ncbi/insdc.sra:SRP180580 (2025).

[101] *NCBI Sequence Read Archive* https://identifiers.org/ncbi/insdc.sra:SRP180579 (2025).

[102] *NCBI Sequence Read Archive* https://identifiers.org/ncbi/insdc.sra:SRP140059 (2025).

[103] *NCBI Sequence Read Archive* https://identifiers.org/ncbi/insdc.sra:SRP140060 (2025).

[104] *NCBI Sequence Read Archive* https://identifiers.org/ncbi/insdc.sra:SRP140070 (2025).

[105] *NCBI Sequence Read Archive* https://identifiers.org/ncbi/insdc.sra:SRP140071 (2025).

[106] *NCBI Sequence Read Archive* https://identifiers.org/ncbi/insdc.sra:SRP140072 (2025).

[107] *NCBI Sequence Read Archive* https://identifiers.org/ncbi/insdc.sra:SRP140073 (2025).

[108] *NCBI Sequence Read Archive* https://identifiers.org/ncbi/insdc.sra:SRP140074 (2025).

[109] *NCBI Sequence Read Archive* https://identifiers.org/ncbi/insdc.sra:SRP140075 (2025).

[110] *NCBI Sequence Read Archive* https://identifiers.org/ncbi/insdc.sra:SRP140076 (2025).

[111] *NCBI Sequence Read Archive* https://identifiers.org/ncbi/insdc.sra:SRP140077 (2025).

[112] *NCBI Sequence Read Archive* https://identifiers.org/ncbi/insdc.sra:SRP140078 (2025).

[113] *NCBI Sequence Read Archive* https://identifiers.org/ncbi/insdc.sra:SRP140080 (2025).

[114] *NCBI Sequence Read Archive* https://identifiers.org/ncbi/insdc.sra:SRP140081 (2025).

[115] *NCBI Sequence Read Archive* https://identifiers.org/ncbi/insdc.sra:SRP140082 (2025).

[116] *NCBI Sequence Read Archive* https://identifiers.org/ncbi/insdc.sra:SRP140083 (2025).

[117] *NCBI Sequence Read Archive* https://identifiers.org/ncbi/insdc.sra:SRP140084 (2025).

[118] *NCBI Sequence Read Archive* https://identifiers.org/ncbi/insdc.sra:SRP140085 (2025).

[119] *NCBI Sequence Read Archive* https://identifiers.org/ncbi/insdc.sra:SRP140086 (2025).

[120] *NCBI Sequence Read Archive* https://identifiers.org/ncbi/insdc.sra:SRP140087 (2025).

[121] *NCBI Sequence Read Archive* https://identifiers.org/ncbi/insdc.sra:SRP140088 (2025).

[122] *NCBI Sequence Read Archive* https://identifiers.org/ncbi/insdc.sra:SRP140089 (2025).

[123] *NCBI Sequence Read Archive* https://identifiers.org/ncbi/insdc.sra:SRP140090 (2025).

[124] *NCBI Sequence Read Archive* https://identifiers.org/ncbi/insdc.sra:SRP140091 (2025).

[125] *NCBI Sequence Read Archive* https://identifiers.org/ncbi/insdc.sra:SRP140092 (2025).

[126] *NCBI Sequence Read Archive* https://identifiers.org/ncbi/insdc.sra:SRP140093 (2025).

[127] *NCBI Sequence Read Archive* https://identifiers.org/ncbi/insdc.sra:SRP140096 (2025).

[128] *NCBI Sequence Read Archive* https://identifiers.org/ncbi/insdc.sra:SRP140097 (2025).

[129] *NCBI Sequence Read Archive* https://identifiers.org/ncbi/insdc.sra:SRP140098 (2025).

[130] *NCBI Sequence Read Archive* https://identifiers.org/ncbi/insdc.sra:SRP140100 (2025).

[131] *NCBI Sequence Read Archive* https://identifiers.org/ncbi/insdc.sra:SRP140101 (2025).

[132] *NCBI Sequence Read Archive* https://identifiers.org/ncbi/insdc.sra:SRP140103 (2025).

[133] *NCBI Sequence Read Archive* https://identifiers.org/ncbi/insdc.sra:SRP140104 (2025).

[134] *NCBI Sequence Read Archive* https://identifiers.org/ncbi/insdc.sra:SRP140105 (2025).

[135] *NCBI Sequence Read Archive* https://identifiers.org/ncbi/insdc.sra:SRP140107 (2025).

[136] *NCBI Sequence Read Archive* https://identifiers.org/ncbi/insdc.sra:SRP140108 (2025).

[137] *NCBI Sequence Read Archive* https://identifiers.org/ncbi/insdc.sra:SRP145620 (2025).

[138] *NCBI Sequence Read Archive* https://identifiers.org/ncbi/insdc.sra:SRP155092 (2025).

[139] *NCBI Sequence Read Archive* https://identifiers.org/ncbi/insdc.sra:SRP155094 (2025).

[140] *NCBI Sequence Read Archive* https://identifiers.org/ncbi/insdc.sra:SRP155096 (2025).

[141] *NCBI Sequence Read Archive* https://identifiers.org/ncbi/insdc.sra:SRP155099 (2025).

[142] *NCBI Sequence Read Archive* https://identifiers.org/ncbi/insdc.sra:SRP155100 (2025).

[143] *NCBI Sequence Read Archive* https://identifiers.org/ncbi/insdc.sra:SRP155101 (2025).

[144] *NCBI Sequence Read Archive* https://identifiers.org/ncbi/insdc.sra:SRP155102 (2025).

[145] *NCBI Sequence Read Archive* https://identifiers.org/ncbi/insdc.sra:SRP155103 (2025).

[146] *NCBI Sequence Read Archive* https://identifiers.org/ncbi/insdc.sra:SRP155107 (2025).

[147] *NCBI Sequence Read Archive* https://identifiers.org/ncbi/insdc.sra:SRP155108 (2025).

[148] *NCBI Sequence Read Archive* https://identifiers.org/ncbi/insdc.sra:SRP155110 (2025).

[149] *NCBI Sequence Read Archive* https://identifiers.org/ncbi/insdc.sra:SRP155111 (2025).

[150] *NCBI Sequence Read Archive* https://identifiers.org/ncbi/insdc.sra:SRP155113 (2025).

[151] *NCBI Sequence Read Archive* https://identifiers.org/ncbi/insdc.sra:SRP155114 (2025).

[152] *NCBI Sequence Read Archive* https://identifiers.org/ncbi/insdc.sra:SRP155115 (2025).

[153] *NCBI Sequence Read Archive* https://identifiers.org/ncbi/insdc.sra:SRP155117 (2025).

[154] *NCBI Sequence Read Archive* https://identifiers.org/ncbi/insdc.sra:SRP155118 (2025).

[155] *NCBI Sequence Read Archive* https://identifiers.org/ncbi/insdc.sra:SRP155119 (2025).

[156] *NCBI Sequence Read Archive* https://identifiers.org/ncbi/insdc.sra:SRP155121 (2025).

[157] *NCBI Sequence Read Archive* https://identifiers.org/ncbi/insdc.sra:SRP155122 (2025).

[158] *NCBI Sequence Read Archive* https://identifiers.org/ncbi/insdc.sra:SRP155126 (2025).

[159] *NCBI Sequence Read Archive* https://identifiers.org/ncbi/insdc.sra:SRP155127 (2025).

[160] *NCBI Sequence Read Archive* https://identifiers.org/ncbi/insdc.sra:SRP155129 (2025).

[161] *NCBI Sequence Read Archive* https://identifiers.org/ncbi/insdc.sra:SRP155130 (2025).

[162] *NCBI Sequence Read Archive* https://identifiers.org/ncbi/insdc.sra:SRP155131 (2025).

[163] *NCBI Sequence Read Archive* https://identifiers.org/ncbi/insdc.sra:SRP155133 (2025).

[164] *NCBI Sequence Read Archive* https://identifiers.org/ncbi/insdc.sra:SRP155135 (2025).

[165] *NCBI Sequence Read Archive* https://identifiers.org/ncbi/insdc.sra:SRP155139 (2025).

[166] *NCBI Sequence Read Archive* https://identifiers.org/ncbi/insdc.sra:SRP155144 (2025).

[167] *NCBI Sequence Read Archive* https://identifiers.org/ncbi/insdc.sra:SRP155145 (2025).

[168] *NCBI Sequence Read Archive* https://identifiers.org/ncbi/insdc.sra:SRP155146 (2025).

[169] *NCBI Sequence Read Archive* https://identifiers.org/ncbi/insdc.sra:SRP155148 (2025).

[170] *NCBI Sequence Read Archive* https://identifiers.org/ncbi/insdc.sra:SRP155149 (2025).

[171] *NCBI Sequence Read Archive* https://identifiers.org/ncbi/insdc.sra:SRP155152 (2025).

[172] *NCBI Sequence Read Archive* https://identifiers.org/ncbi/insdc.sra:SRP155153 (2025).

[173] *NCBI Sequence Read Archive* https://identifiers.org/ncbi/insdc.sra:SRP155154 (2025).

[174] *NCBI Sequence Read Archive* https://identifiers.org/ncbi/insdc.sra:SRP155158 (2025).

[175] *NCBI Sequence Read Archive* https://identifiers.org/ncbi/insdc.sra:SRP155159 (2025).

[176] *NCBI Sequence Read Archive* https://identifiers.org/ncbi/insdc.sra:SRP155160 (2025).

[177] *NCBI Sequence Read Archive* https://identifiers.org/ncbi/insdc.sra:SRP155161 (2025).

[178] *NCBI Sequence Read Archive* https://identifiers.org/ncbi/insdc.sra:SRP183817 (2025).

[179] *NCBI Sequence Read Archive* https://identifiers.org/ncbi/insdc.sra:SRP194746 (2025).

[180] *NCBI Sequence Read Archive* https://identifiers.org/ncbi/insdc.sra:SRP194747 (2025).

[181] *NCBI Sequence Read Archive* https://identifiers.org/ncbi/insdc.sra:SRP194749 (2025).

[182] *NCBI Sequence Read Archive* https://identifiers.org/ncbi/insdc.sra:SRP184580 (2025).

[183] *NCBI Sequence Read Archive* https://identifiers.org/ncbi/insdc.sra:SRP184576 (2025).

[184] *NCBI Sequence Read Archive* https://identifiers.org/ncbi/insdc.sra:SRP184568 (2025).

[185] *NCBI Sequence Read Archive* https://identifiers.org/ncbi/insdc.sra:SRP184571 (2025).

[186] *NCBI Sequence Read Archive* https://identifiers.org/ncbi/insdc.sra:SRP184582 (2025).

[187] *NCBI Sequence Read Archive* https://identifiers.org/ncbi/insdc.sra:SRP184572 (2025).

[188] *NCBI Sequence Read Archive* https://identifiers.org/ncbi/insdc.sra:SRP184579 (2025).

[189] *NCBI Sequence Read Archive* https://identifiers.org/ncbi/insdc.sra:SRP184575 (2025).

[190] *NCBI Sequence Read Archive* https://identifiers.org/ncbi/insdc.sra:SRP184570 (2025).

[191] *NCBI Sequence Read Archive* https://identifiers.org/ncbi/insdc.sra:SRP184574 (2025).

[192] *NCBI Sequence Read Archive* https://identifiers.org/ncbi/insdc.sra:SRP184551 (2025).

[193] *NCBI Sequence Read Archive* https://identifiers.org/ncbi/insdc.sra:SRP184552 (2025).

[194] *NCBI Sequence Read Archive* https://identifiers.org/ncbi/insdc.sra:SRP184559 (2025).

[195] *NCBI Sequence Read Archive* https://identifiers.org/ncbi/insdc.sra:SRP184562 (2025).

[196] *NCBI Sequence Read Archive* https://identifiers.org/ncbi/insdc.sra:SRP184564 (2025).

[197] *NCBI Sequence Read Archive* https://identifiers.org/ncbi/insdc.sra:SRP184560 (2025).

[198] *NCBI Sequence Read Archive* https://identifiers.org/ncbi/insdc.sra:SRP184561 (2025).

[199] *NCBI Sequence Read Archive* https://identifiers.org/ncbi/insdc.sra:SRP184563 (2025).

[200] *NCBI Sequence Read Archive* https://identifiers.org/ncbi/insdc.sra:SRP184565 (2025).

[201] *NCBI Sequence Read Archive* https://identifiers.org/ncbi/insdc.sra:SRP184566 (2025).

[202] *NCBI Sequence Read Archive* https://identifiers.org/ncbi/insdc.sra:SRP197169 (2025).

[203] *NCBI Sequence Read Archive* https://identifiers.org/ncbi/insdc.sra:SRP197170 (2025).

[204] *NCBI Sequence Read Archive* https://identifiers.org/ncbi/insdc.sra:SRP197165 (2025).

[205] *NCBI Sequence Read Archive* https://identifiers.org/ncbi/insdc.sra:SRP197173 (2025).

[206] *NCBI Sequence Read Archive* https://identifiers.org/ncbi/insdc.sra:SRP197167 (2025).

[207] *NCBI Sequence Read Archive* https://identifiers.org/ncbi/insdc.sra:SRP197160 (2025).

[208] *NCBI Sequence Read Archive* https://identifiers.org/ncbi/insdc.sra:SRP257045 (2025).

[209] *NCBI Sequence Read Archive* https://identifiers.org/ncbi/insdc.sra:SRP197166 (2025).

[210] *NCBI Sequence Read Archive* https://identifiers.org/ncbi/insdc.sra:SRP257042 (2025).

[211] *NCBI Sequence Read Archive* https://identifiers.org/ncbi/insdc.sra:SRP197174 (2025).

[212] *NCBI Sequence Read Archive* https://identifiers.org/ncbi/insdc.sra:SRP197155 (2025).

[213] *NCBI Sequence Read Archive* https://identifiers.org/ncbi/insdc.sra:SRP197157 (2025).

[214] *NCBI Sequence Read Archive* https://identifiers.org/ncbi/insdc.sra:SRP197156 (2025).

[215] *NCBI Sequence Read Archive* https://identifiers.org/ncbi/insdc.sra:SRP257044 (2025).

[216] *NCBI Sequence Read Archive* https://identifiers.org/ncbi/insdc.sra:SRP197158 (2025).

[217] *NCBI Sequence Read Archive* https://identifiers.org/ncbi/insdc.sra:SRP197153 (2025).

[218] *NCBI Sequence Read Archive* https://identifiers.org/ncbi/insdc.sra:SRP197161 (2025).

[219] *NCBI Sequence Read Archive* https://identifiers.org/ncbi/insdc.sra:SRP197159 (2025).

[220] *NCBI Sequence Read Archive* https://identifiers.org/ncbi/insdc.sra:SRP257043 (2025).

[221] *NCBI Sequence Read Archive* https://identifiers.org/ncbi/insdc.sra:SRP257046 (2025).

[222] *NCBI Sequence Read Archive* https://identifiers.org/ncbi/insdc.sra:SRP184595 (2025).

[223] *NCBI Sequence Read Archive* https://identifiers.org/ncbi/insdc.sra:SRP184592 (2025).

[224] *NCBI Sequence Read Archive* https://identifiers.org/ncbi/insdc.sra:SRP184599 (2025).

[225] *NCBI Sequence Read Archive* https://identifiers.org/ncbi/insdc.sra:SRP184602 (2025).

[226] *NCBI Sequence Read Archive* https://identifiers.org/ncbi/insdc.sra:SRP184603 (2025).

[227] *NCBI Sequence Read Archive* https://identifiers.org/ncbi/insdc.sra:SRP184598 (2025).

[228] *NCBI Sequence Read Archive* https://identifiers.org/ncbi/insdc.sra:SRP184596 (2025).

[229] *NCBI Sequence Read Archive* https://identifiers.org/ncbi/insdc.sra:SRP184597 (2025).

[230] *NCBI Sequence Read Archive* https://identifiers.org/ncbi/insdc.sra:SRP184594 (2025).

[231] *NCBI Sequence Read Archive* https://identifiers.org/ncbi/insdc.sra:SRP184593 (2025).

[232] *NCBI Sequence Read Archive* https://identifiers.org/ncbi/insdc.sra:SRP184581 (2025).

[233] *NCBI Sequence Read Archive* https://identifiers.org/ncbi/insdc.sra:SRP184586 (2025).

[234] *NCBI Sequence Read Archive* https://identifiers.org/ncbi/insdc.sra:SRP184584 (2025).

[235] *NCBI Sequence Read Archive* https://identifiers.org/ncbi/insdc.sra:SRP184583 (2025).

[236] *NCBI Sequence Read Archive* https://identifiers.org/ncbi/insdc.sra:SRP184587 (2025).

[237] *NCBI Sequence Read Archive* https://identifiers.org/ncbi/insdc.sra:SRP184585 (2025).

[238] *NCBI Sequence Read Archive* https://identifiers.org/ncbi/insdc.sra:SRP184588 (2025).

[239] *NCBI Sequence Read Archive* https://identifiers.org/ncbi/insdc.sra:SRP184589 (2025).

[240] *NCBI Sequence Read Archive* https://identifiers.org/ncbi/insdc.sra:SRP184590 (2025).

[241] *NCBI Sequence Read Archive* https://identifiers.org/ncbi/insdc.sra:SRP184591 (2025).

[242] *NCBI Sequence Read Archive* https://identifiers.org/ncbi/insdc.sra:SRP184623 (2025).

[243] *NCBI Sequence Read Archive* https://identifiers.org/ncbi/insdc.sra:SRP184624 (2025).

[244] *NCBI Sequence Read Archive* https://identifiers.org/ncbi/insdc.sra:SRP184627 (2025).

[245] *NCBI Sequence Read Archive* https://identifiers.org/ncbi/insdc.sra:SRP256937 (2025).

[246] *NCBI Sequence Read Archive* https://identifiers.org/ncbi/insdc.sra:SRP184625 (2025).

[247] *NCBI Sequence Read Archive* https://identifiers.org/ncbi/insdc.sra:SRP184621 (2025).

[248] *NCBI Sequence Read Archive* https://identifiers.org/ncbi/insdc.sra:SRP184619 (2025).

[249] *NCBI Sequence Read Archive* https://identifiers.org/ncbi/insdc.sra:SRP184618 (2025).

[250] *NCBI Sequence Read Archive* https://identifiers.org/ncbi/insdc.sra:SRP184620 (2025).

[251] *NCBI Sequence Read Archive* https://identifiers.org/ncbi/insdc.sra:SRP184617 (2025).

[252] *NCBI Sequence Read Archive* https://identifiers.org/ncbi/insdc.sra:SRP184609 (2025).

[253] *NCBI Sequence Read Archive* https://identifiers.org/ncbi/insdc.sra:SRP184607 (2025).

[254] *NCBI Sequence Read Archive* https://identifiers.org/ncbi/insdc.sra:SRP184608 (2025).

[255] *NCBI Sequence Read Archive* https://identifiers.org/ncbi/insdc.sra:SRP184606 (2025).

[256] *NCBI Sequence Read Archive* https://identifiers.org/ncbi/insdc.sra:SRP184605 (2025).

[257] *NCBI Sequence Read Archive* https://identifiers.org/ncbi/insdc.sra:SRP184604 (2025).

[258] *NCBI Sequence Read Archive* https://identifiers.org/ncbi/insdc.sra:SRP184610 (2025).

[259] *NCBI Sequence Read Archive* https://identifiers.org/ncbi/insdc.sra:SRP184613 (2025).

[260] *NCBI Sequence Read Archive* https://identifiers.org/ncbi/insdc.sra:SRP184612 (2025).

[261] *NCBI Sequence Read Archive* https://identifiers.org/ncbi/insdc.sra:SRP184615 (2025).

[262] NCBI Genbank https://identifiers.org/ncbi/insdc:JBTAFJ000000000 (2026).

[263] NCBI Genbank https://identifiers.org/ncbi/insdc:JBTIYH000000000 (2026).

[264] NCBI Genbank https://identifiers.org/ncbi/insdc:JBTYLQ000000000 (2026).

[265] NCBI Genbank https://identifiers.org/ncbi/insdc:JBTYLS000000000 (2026).

[266] NCBI Genbank https://identifiers.org/ncbi/insdc:JBTYLR000000000 (2026).

[267] NCBI Genbank https://identifiers.org/ncbi/insdc:WHVC00000000 (2026).

[268] NCBI Genbank https://identifiers.org/ncbi/insdc:WIQF00000000 (2026).

[269] NCBI Genbank https://identifiers.org/ncbi/insdc:WION00000000 (2026).

[270] NCBI Genbank https://identifiers.org/ncbi/insdc:WHVB00000000 (2026).

[271] Callahan, B. J. *et al*. DADA2: High-resolution sample inference from Illumina amplicon data. *Nat. Methods* **13**, 581–583, 10.1038/nmeth.3869 (2016). 27214047 10.1038/nmeth.3869 PMC4927377

[272] Větrovský, T., Baldrian, P. & Morais, D. SEED 2: a user-friendly platform for amplicon high-throughput sequencing data analyses. *Bioinformatics* **34**, 2292–2294, 10.1093/bioinformatics/bty071 (2018). 29452334 10.1093/bioinformatics/bty071 PMC6022770

[273] Edgar, R. C. Search and clustering orders of magnitude faster than BLAST. *Bioinformatics* **26**, 2460–2461, 10.1093/bioinformatics/btq461 (2010). 20709691 10.1093/bioinformatics/btq461

[274] GOLD Study Gs0128948. [https://gold.jgi.doe.gov/study?id=Gs0128948].

[275] Chen, I.-A. *et al*. IMG/M v.5.0: an integrated data management and comparative analysis system for microbial genomes and microbiomes. *Nucleic Acids Res.* **47**, D666–D677, 10.1093/nar/gky901 (2019). 30289528 10.1093/nar/gky901 PMC6323987

[276] IMG/M ER. [https://img.jgi.doe.gov/mer/].

[277] MycoCosm Home. [https://mycocosm.jgi.doe.gov/mycocosm/home].

[278] ElagrMar1 genome. [https://mycocosm.jgi.doe.gov/ElagrMar1/ElagrMar1.home.html].

[279] Lactab1 genome. [https://mycocosm.jgi.doe.gov/Lactab1/Lactab1.home.html].

[280] RusdecM1 genome. [https://mycocosm.jgi.doe.gov/RusdecM1/RusdecM1.home.html].

[281] Tylfel1 genome. [https://mycocosm.jgi.doe.gov/Tylfel1/Tylfel1.home.html].

[282] Hyavar1 genome. [https://mycocosm.jgi.doe.gov/Hyavar1/Hyavar1.home.html].

[283] Ruseme1 genome. [https://mycocosm.jgi.doe.gov/Ruseme1/Ruseme1.home.html].

[284] Boledp1 genome. [https://mycocosm.jgi.doe.gov/Boledp1/Boledp1.home.html].

[285] Amarub1 genome. [https://mycocosm.jgi.doe.gov/Amarub1/Amarub1.home.html].

[286] Rusoch1 genome. [https://mycocosm.jgi.doe.gov/Rusoch1/Rusoch1.home.html].

